# Non-rheumatic Streptococcal Acute Myocarditis: The First Documented Sibling Case

**DOI:** 10.7759/cureus.60990

**Published:** 2024-05-24

**Authors:** Batoul Mcheik, Hassan B Ayach, Georgio El Koubayati, Rim M Abdallah, Majd Khalil, Mouin Jammal, Fady Haddad, Naji Abi Rached

**Affiliations:** 1 Internal Medicine, Lebanese University Faculty of Medicine, Beirut, LBN; 2 Cardiology, Lebanese University Faculty of Medicine, Beirut, LBN; 3 Internal Medicine and Clinical Immunology, Lebanese University Faculty of Medicine, Beirut, LBN; 4 Cardiology, Lebanese Hospital Geitaoui, Beirut, LBN; 5 Internal Medicine and Clinical Immunology, Lebanese Hospital Geitaoui, Beirut, LBN

**Keywords:** non-rheumatic streptococcal acute myocarditis, spam, infectious myocarditis, group a β-hemolytic streptococcus pyogenes, myocarditis

## Abstract

Myocarditis is an inflammatory disease of the cardiac muscle that manifests as chest pain, dyspnea, and other signs of heart failure. ST segment changes with elevated cardiac biomarkers mimic acute coronary syndromes. It is most commonly caused by viruses like the Epstein-Barr virus (EBV) and Coxsackie B virus, but it can also be due to cardiotoxic drugs like cyclophosphamide and cocaine or caused by a systemic infiltrative process like sarcoidosis or collagen vascular diseases. One relatively common bacterial cause of myocarditis is beta-hemolytic Group A *Streptococcus*, which is well known to lead, two to three weeks later, to rheumatic fever and pancarditis. Less commonly, it can cause non-rheumatic myocarditis, which occurs faster, with the pathogenesis not very well understood.

We will be reporting a case series of two brothers suffering at the same time from non-rheumatic streptococcal A-isolated myocarditis, questioning the possibility of bacterial toxin-mediated myocarditis or inter-linked genetic predisposition.

## Introduction

Myocarditis is an entity characterized by inflammation, thickness, and eventually failure of the heart muscle. It is commonly caused by a viral infection such as adenovirus or COVID-19. Moreover, organisms such as *Staphylococcus*, *Streptococcus*, *Corynebacterium diphtheriae*, *Trypanosoma cruzi*, *Toxoplasma gondii*, and certain fungi may lead to myocarditis. Other rare causes include autoimmune diseases, vasculitis, and chronic inflammatory conditions like sarcoidosis, granulomatosis with polyangiitis, and thyrotoxicosis. Symptoms may vary from atypical chest discomfort to frank signs of heart failure and circulatory collapse.

Non-rheumatic streptococcal acute myocarditis, also known as strep pharyngitis acute myocarditis (SPAM), is more prevalent in males and appears in young people [1-5]. It is characterized by ECG changes revealing ST segment changes, similar to alterations seen in acute myocardial infarction (AMI) [4], but not meeting the criteria of rheumatic fever [6]. The symptoms of SPAM usually appear within five days of the original streptococcal illness. On the other hand, myocarditis in rheumatic fever caused by group A streptococcal (GAS) infection typically appears after a two- to three-week interval of latent infection [7]. It is worth noting that acute rheumatic fever (ARF) affects the entire heart (i.e., pericardium, epicardium, myocardium, and endocardium) and causes mostly valvular disease. [8]

In the following article, we will report the case of SPAM occurring simultaneously in two young siblings.

## Case presentation

We present the cases of two male siblings, aged 21 and 24, who presented with chest pain and tonsillitis to the Lebanese Hospital Geitaoui, University Medical Center.

**Figure 1:**
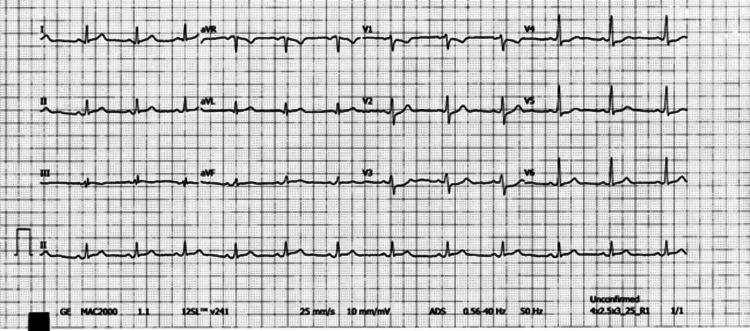
The ECG of the younger sibling (a 21-year-old male) showed non-specific ST depression through the precordial leads.

A previously healthy 21-year-old man presented to the emergency department with substernal chest pain radiating to his left arm associated with nausea, dyspnea, sore throat, and ear pain. Two days before the presentation, he was evaluated at local urgent care for a sore throat and tonsillar erythema and was started on oral amoxicillin with little to no improvement in his symptoms. Physical examination revealed a regular rate and rhythm without any murmurs, rubs, or gallops. Lungs were clear to auscultation. The ECG showed non-specific ST depression through the precordial leads (Figure 1).

The patient's labs on presentation are shown in Table 1.

**Table 1:** Laboratory results of the first sibling showed leukocytosis and elevated cardiac and muscle enzymes.

Laboratory exam	Patient’s results	Reference range
Hemoglobin	12.9	12-16 g/dL
Mean corpuscular volume	91	81-99 fl
White blood cells	13,900	4,800-10,800/mm3
Troponin T levels	617	0-16 ng/L
Creatine phosphokinase (CK)	470	26-174U/L
CK-myocardial band (MB)	39	0-3 ng/mL

**Figure 2:**
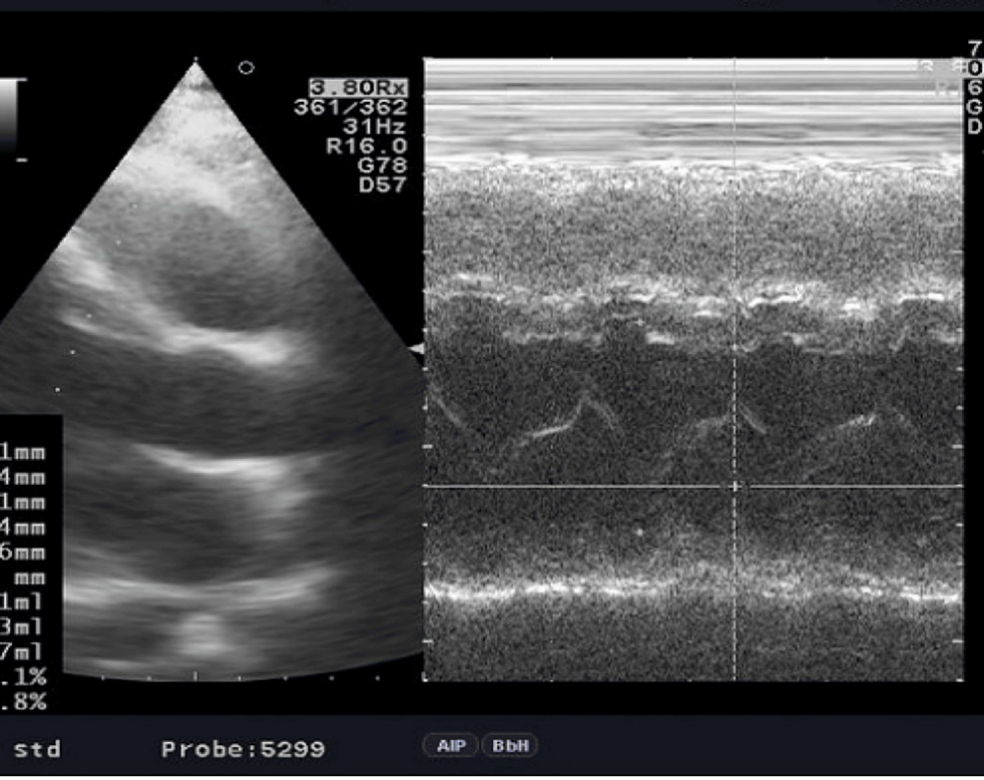
A transthoracic echocardiogram (TTE) of the first sibling showed apical hypokinesis.

They were significant for troponin T levels of 617 ng/L (reference range: 0-16 ng/L), creatine phosphokinase (CK) of 470 U/L (reference range: 26-174 U/L), and CK-myocardial band (MB) of 39 ng/mL (reference range: 0-3 ng/mL). Other labs done showed leukocytosis of 13.900/mm3 with a left shift in neutrophils. An urgent transthoracic echocardiogram (TTE) showed mild apical hypokinesis (Figure 2).

The second day, troponin T levels significantly increased to 2019 ng/L, CK levels more than doubled to reach 947 U/L, and CK-MB almost tripled to 101 ng/mL. Cytomegalovirus (CMV), Epstein-Barr virus (EBV), and mycoplasma IgG levels were positive, but IgM turned negative, ruling out any acute infectious process. However, a rapid strep A test turned out to be positive, and throat culture grew *Streptococcus pyogenes. *

**Figure 3:**
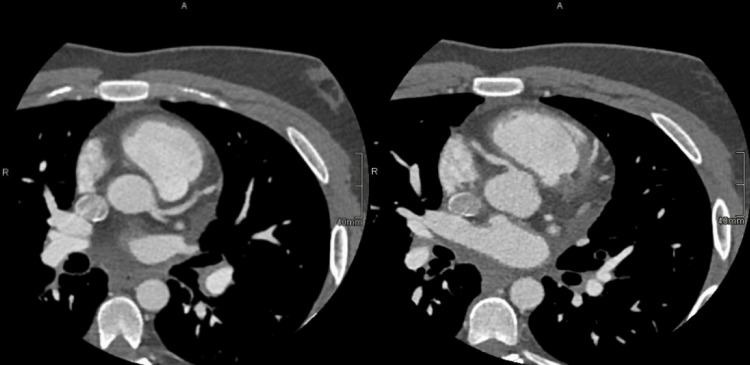
Axial view of the coronary CT angiogram (Coro-scan) of the younger sibling showed normal and patent coronary arteries, favoring the diagnosis of myocarditis.

The coronary CT angiogram was unremarkable, favoring the diagnosis of post-bacterial myocarditis (Figure 3).

Based on ST segment changes, since elevated cardiac biomarkers, normal angiography, and failure to meet the Revised Jones Criteria for diagnosis of acute rheumatic fever (ARF), the patient was diagnosed with SPAM. He was treated with a 10-day course of ceftriaxone 2 mg IV once daily and clindamycin 600 mg IV q8h, non-steroidal anti-inflammatory drugs (naproxen 250 mg per oral (PO) every 12 hours (q12h)) for 14 days, and a six-month regimen of angiotensin-converting enzyme (ACE) inhibitor (ramipril 5 mg PO once a day (OD)), beta-blocker (bisoprolol 2.5 mg PO q12h), and aldosterone receptor antagonist (eplerenone 25 mg PO OD). The symptoms started to improve rapidly a few days later. The patient was also given instructions to refrain from strenuous physical activity for six months.

The older brother, who is a 24-year-old active, healthy male, smoker, and non-alcoholic, presented to the emergency department the day after his brother’s presentation with the same symptoms, which were as follows: exertional chest discomfort and pleuritic pain associated with shortness of breath. These manifestations had begun two days following pharyngitis, for which he was treated with penicillin. Vitals upon presentation were normal. 

**Figure 4:**
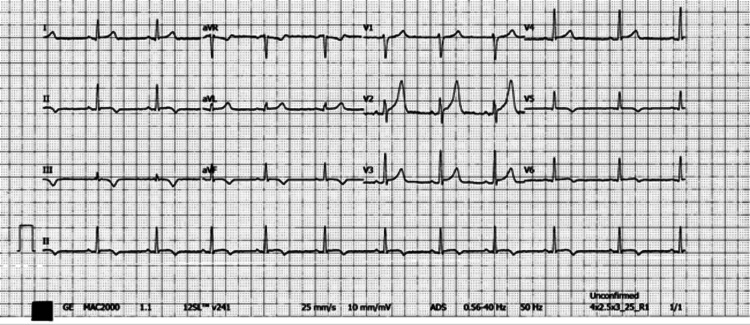
The 12-lead ECG of the 24-year-old sibling showed diffuse ST elevation (precordial leads) and T wave inversions as dynamic changes (chest leads).

The ECG demonstrated diffuse ST elevation followed by T wave inversions as dynamic changes (Figure 4).

**Figure 5:**
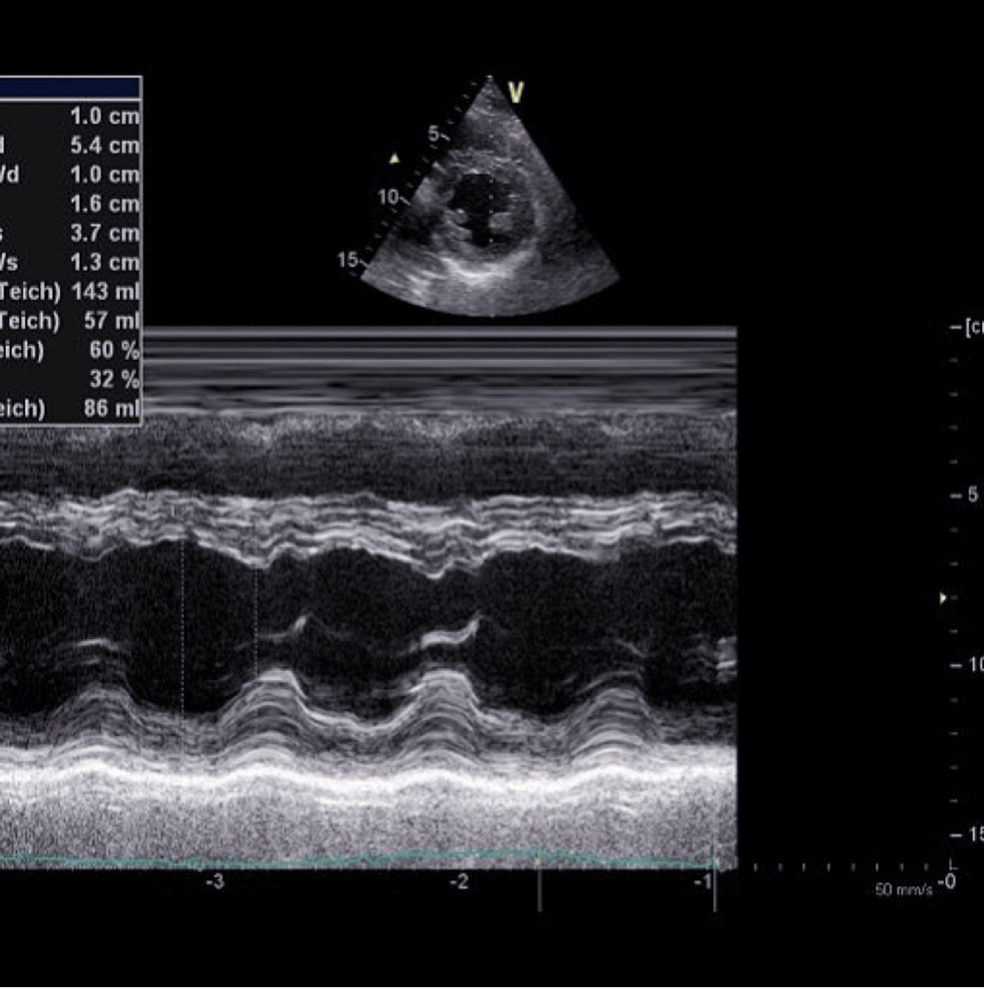
The TTE of the older sibling showed global hypokinesia with reduced LVEF reaching 35%. TTE: transthoracic echocardiogram; LVEF: left ventricular ejection fraction

Troponin levels showed an incremental rise from 460 to 727 ng/L. The CMV and EBV tests were negative, but the throat swab and throat culture for GAS tested positive. Urgent TEE showed global hypokinesia with a reduced left ventricular ejection fraction (LVEF) reaching 35% (Figure 5).

**Figure 6:**
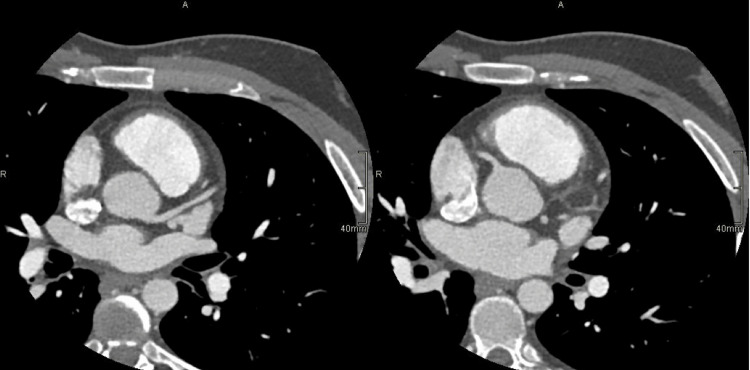
A coro-scan of the older sibling showed normal and patent coronaries, confirming the diagnosis of myocarditis.

The Coro-scan revealed normal coronaries (Figure 6), and inflammatory markers (erythrocyte sedimentation rate (ESR) and C-reactive protein (CRP)) were elevated. Consequently, myocarditis was the primary diagnosis.

He received optimal medical therapy for heart failure for six months including beta-blockers, ACE inhibitors, aldosterone receptor antagonists, sodium-glucose co-transporter-2 (SGLT2) inhibitors (dapagliflozin 10mg PO OD), and diuretics. During follow-up, ECG and echocardiography were normalized. Repeated echocardiography within three weeks demonstrated normal left ventricle function with an ejection fraction (EF) of 60%.

## Discussion

In most cases, myocarditis following GAS pharyngitis is attributed to ARF, which manifests itself two to four weeks after the bacterial infection and is associated with other symptoms such as pancarditis (50%-78%), mainly valvulitis, arthritis (35%-88%), erythema marginatum (6%), and subcutaneous nodules (1%-13%) [9]. Moreover, Sydenham's chorea, a neurological illness marked by uncontrollable movements and behavioral abnormalities, is present in 2%-19% of patients [9]; in our cases, both patients didn’t show these features. On the other hand, numerous examples suggest that myocarditis can develop shortly after the streptococcal infection and is confirmed by a rapid antigen test or culture with concomitant exclusion of the ARF criteria and normal coronaries on angio-coronary CT, favoring the diagnosis of SPAM. [7]

This condition has been referred to as non-rheumatic myocarditis since Gore and Saphir were the first to describe it in 1947. It occurs mainly in young patients, primarily men, and typically presents with non-pleuritic chest discomfort, sporadic fever, and this type of myocarditis [9]. Most patients described in the literature fully recovered in a short period (days to weeks) with no sequelae.

Eosinophilic hypersensitivity myocarditis (EM) could be one of the differentials in these two patients and might be induced by the treatment of the disease with penicillin. However, it is less reasonable because these patients' blood counts demonstrated neither eosinophilia (3% in patient #1, 2% in patient #2) nor clinical signs of hypersensitivity reactions. Eosinophilic hypersensitivity myocarditis is often fatal and usually fulminant. Endomyocardial biopsy permits a definitive diagnosis [10]. Moreover, clinical symptoms, ECG changes, and cardiac ultrasound favored the diagnosis of myocarditis related to bacterial pharyngitis, for which adequate treatment was started, resulting in rapid recovery.

In our study, we reported a case series of two brothers suffering simultaneously from SPAM myocarditis. Although no literature review about the genetic predisposition of infective myocarditis has been reported, except for dilated cardiomyopathy [11], two cases of monozygotic twins after the second dose of the COVID-19 vaccine [12] were published. These reported cases suggested the involvement of genetic factors in the development of post-infectious or post-vaccine-induced myocarditis.

Consequently, two hypotheses were discussed in the literature. Streptococcal toxins and cross-reactivity have been proposed as potential causal pathways for the development of non-rheumatic myocarditis following streptococcal pharyngitis or tonsillitis. The poison theory (toxin-mediated), however, appears to be more likely to be accepted, as shown in the study by Gore and Saphir, which could support this assumption simply because the same germ causes the same disease in two different patients, such as brothers that are not genetically identical [13]. Hence, the relationship between pathogenesis and genetic predisposition needs to be better clarified later on. A recent study by Hiraiwa et al. [14] documented a patient with recurrent myocarditis whose autopsy and endomyocardial biopsies revealed neutrophilic infiltration, favoring the cross-reactivity theory. Moreover, small abscesses with nearby germs were discovered.

According to various case reports and follow-ups with our patients, penicillin remains the first-line antimicrobial treatment for streptococcal pharyngitis. Cephalosporins and erythromycin are substitutes for those with severe penicillin allergies [15]. Although corticosteroids remain an option for people with contraindications such as hemorrhage, kidney failure, and others, non-steroidal anti-inflammatory drugs (such as naproxen sodium and ibuprofen) should be used as the first-line treatment against inflammation. 

Such a category of cardiomyopathies in these patients is typically self-limited. Treatment options using beta-blockers, ACE/angiotensin receptor blockers (ARB) inhibitors, aldosterone receptor antagonists, SGLT2 inhibitors, and diuretics (for volume control) are mandatory if features of heart failure are settled.

It is worth mentioning that cardiac magnetic resonance imaging remains one of the essential modalities for identifying suspected myocardial inflammation with subsequent edema and injury. Thus, it helps in differentiating the etiologies and guiding future therapies.

## Conclusions

This article presents a rare case of simultaneous SPAM in two siblings. It occurs earlier than myocarditis in rheumatic fever but can lead to significant morbidity and mortality if not recognized and treated accordingly. The relationship between the pathogenesis of SPAM and genetic predisposition needs to be further clarified with further studies.
